# An Overview of In Vitro Assays of ^64^Cu-, ^68^Ga-, ^125^I-, and ^99m^Tc-Labelled Radiopharmaceuticals Using Radiometric Counters in the Era of Radiotheranostics

**DOI:** 10.3390/diagnostics13071210

**Published:** 2023-03-23

**Authors:** Viviana Benfante, Alessandro Stefano, Muhammad Ali, Riccardo Laudicella, Walter Arancio, Antonino Cucchiara, Fabio Caruso, Francesco Paolo Cammarata, Claudia Coronnello, Giorgio Russo, Monica Miele, Alessandra Vieni, Antonino Tuttolomondo, Anthony Yezzi, Albert Comelli

**Affiliations:** 1 Ri.MED Foundation, Via Bandiera 11, 90133 Palermo, Italyacomelli@fondazionerimed.com (A.C.); 2 Department of Health Promotion, Mother and Child Care, Internal Medicine and Medical Specialties, Molecular and Clinical Medicine, University of Palermo, 90127 Palermo, Italy; 3 Institute of Molecular Bioimaging and Physiology, National Research Council (IBFM-CNR), 90015 Cefalù, Italy; 4 Department of Diagnostic and Therapeutic Services, IRCCS-ISMETT (Mediterranean Institute for Transplantation and Advanced Specialized Therapies), Via Tricomi 5, 90127 Palermo, Italy; 5 National Biodiversity Future Center (NBFC), 90133 Palermo, Italy; 6 Department of Electrical and Computer Engineering, Georgia Institute of Technology, Atlanta, GA 30332, USA

**Keywords:** in vitro test, radiopharmaceuticals radiobiology, gamma counter, radiotheranostics, imaging, cancer

## Abstract

Radionuclides are unstable isotopes that mainly emit alpha (α), beta (β) or gamma (γ) radiation through radiation decay. Therefore, they are used in the biomedical field to label biomolecules or drugs for diagnostic imaging applications, such as positron emission tomography (PET) and/or single-photon emission computed tomography (SPECT). A growing field of research is the development of new radiopharmaceuticals for use in cancer treatments. Preclinical studies are the gold standard for translational research. Specifically, in vitro radiopharmaceutical studies are based on the use of radiopharmaceuticals directly on cells. To date, radiometric β- and γ-counters are the only tools able to assess a preclinical in vitro assay with the aim of estimating uptake, retention, and release parameters, including time- and dose-dependent cytotoxicity and kinetic parameters. This review has been designed for researchers, such as biologists and biotechnologists, who would like to approach the radiobiology field and conduct in vitro assays for cellular radioactivity evaluations using radiometric counters. To demonstrate the importance of in vitro radiopharmaceutical assays using radiometric counters with a view to radiogenomics, many studies based on ^64^Cu-, ^68^Ga-, ^125^I-, and ^99m^Tc-labeled radiopharmaceuticals have been revised and summarized in this manuscript.

## 1. Introduction

Targeted radionuclide therapy (TRT) is a branch of nuclear medicine based on the cytotoxic and genotoxic effects generated by radiation on a specific biological target. While in classical radiotherapy, the target is a body part or a spatially defined portion of tissue, in TRT, a specific portion of cancer cells, such as a receptor, represents the target. TRT is based on the accumulation of the radiolabeled compound on target cells, causing a certain degree of short-range cytotoxicity and genotoxicity by ionizing radiation emitted via the radionuclide. Several factors have contributed to the development of TRT-targeting molecules, including the type of emission, the physical half-life, linear energy transfer (LET), as well as biochemical reactions occurring in cells.

All of these aspects are studied in radiobiology [1], a branch of biology closely related to physics. Applied to the medical field, radiobiology examines the effects of ionizing radiation (produced by radionuclides or charged particle beams) on cells and tissues, aiming to optimize dose administration, and minimize risks while maximizing benefits. As well as studying the internalization and externalization kinetics of drugs within cells, the mechanism of activation of specific biochemical pathways, as well as changes in cellular morphology, are also explored in this research. Furthermore, the creation of a standard in vitro model, using cells, is essential for correlating dose-related effects and biological endpoints, in order to highlight the risks and benefits of radioactivity exposure to radiopharmaceuticals. 

The study of the genomic changes that underlie the radioreceptive response of normal and tumor tissues is called radiogenomics [2]. 

**Figure 1:**
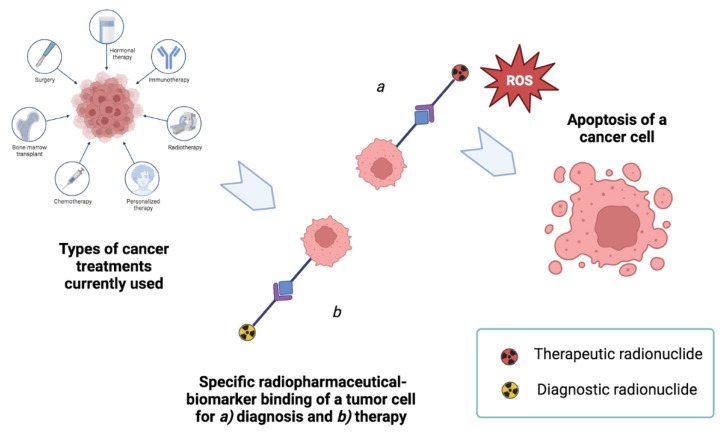
The concept of radiotheranostics within the framework of treatments used in case of cancer disease. In this case, the objective of the research is to develop pharmaceutical formulations that keep the radionuclide component unchanged, but adapt the ligand component according to its (a) diagnostic or (b) therapeutic purpose.

A fundamental research field in the modern era is radiopharmaceutical theranostics (radiotheranostics) [3], a branch of research focused on the discovery of innovative radiopharmaceuticals for a double approach [4], that allows the use of a single radioisotope or a pair of radionuclides to mark a single target vector, usable both for diagnostic tracer-imaging (e.g., by unstable γ-emitting or β+-emitting nuclides) and for therapy treatment as drugs (e.g., by unstable β−-emitting nuclides) (see Figure 1) [5,6]. Thus, studies on the efficacy of ionizing radiation on tissues provide a better understanding of the interactions between receptors and molecule vectors selected for radiopharmaceuticals. Additionally, new knowledge of the impact of dose-response relationships on cellular models is gaining recognition, enabling procedures to become translational and standard within clinical practice, which is not yet true in the modern world [7,8].

Considering biologists and biotechnologists as the target audience, this publication has been structured in such a way as to offer an ordered overview of the various basic issues useful for understanding this review, in order to guide the reader to a multidisciplinary path, from imaging techniques to radiotheranostics [3], which are closely related to nuclear medicine, radiopharmaceutical radiobiology [9], physics [10], radiochemistry [11], artificial intelligence (machine learning and deep learning), radiomics techniques [12], and radiogenomics [13]. The reader will be directed to various works in which the use of a γ-counter for in vitro radiometric assays is considered. These assays are used for the evaluation of radioactivity in some cell lines. It should be noted that the radiometric measurements and in vitro tests referred to throughout the review do not take into consideration the quality control understood as Good Manufacturing Practice (GMP), but aim to introduce the case for radioactivity measurement using radiometric counters and two-dimensional cell cultures treated with radiopharmaceuticals, that can be carried out in carefully equipped research laboratories with radiation protection.

Section 2 describes radionuclide classification, while Section 3 discusses the use of radiopharmaceuticals with imaging techniques and radiometric counters. Section 3 is divided into two subsections, namely (3.1) the β counter, and (3.2) the γ counter. Section 4 describes the use of the γ counter for in vitro tests, with the following radionuclides: (4.1) copper-64 (^64^Cu), (4.2) gallium-68 (^68^Ga), (4.3) iodine-125 (^125^I), and (4.4) technetium-99 metastable (^99m^Tc).

## 2. Classification of Radionuclides and New Applications in Medical Research

Radionuclides are unstable nuclides, which, emitting α, β^+/−^ charged particles, Auger electrons, and/or γ rays [14,15,16] through radioactive decay processes, are commonly used in the medical field, both for diagnosis and therapy purposes [14].

Radionuclides for diagnostic use can be classified into two types: (1) radionuclides that emit almost exclusively β^+^ rays (detectable through positron emission tomography—PET), and (2) γ rays (detectable through single-photon emission computed tomography—SPECT). On the contrary, radionuclides used for therapeutics are characterized by α, β^-^, or Auger electron decay [9]. For the assessment of the biological efficacy of the radiopharmaceutical dose on specific targets, some aspects must be considered. To use radionuclides for therapy, scientists selected properties, such as half-life, energy, and activity of the nuclides to balance the effects of radiosensitization on the target tumor, avoiding the adaptive responses characteristic of cancerous tissues, so as to enhance tumor ablation and decrease tumor radioresistance mechanisms.

**Figure 2:**
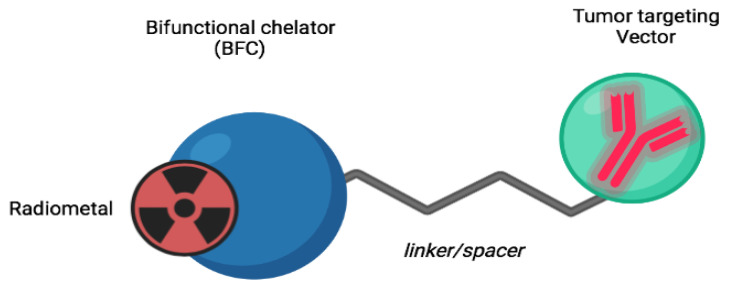
The design of a canonical radiopharmaceutical compound. A radiometal represents a specific radionuclide. BFC stands for a chemical compound that chelates radionuclides; a linker/spacer is a link between two molecules; the tumor-targeting vector recognizes and binds a tumor biomarker to a cell.

Two primary components characterize radiopharmaceuticals: (i) a radionuclide that confers tracing or damaging properties to the molecule by ionizing radiation, and (ii) a specific biomolecule or ligand defining the radiopharmaceutical binding and uptake of cell targets, as well as two additional significant components, a chelator and a linker, which ensure drug chemical stability (see Figure 2). Contrary to radiopharmaceuticals containing radionuclides such as iodine, fluorine, or carbon isotopes that are covalently attached to carriers, most imaging radiopharmaceuticals contain a metal as part of their design. As a result of the presence of the metal, high thermodynamic, and kinetic stability is required to avoid transmetallation reactions, i.e., the metal binds to other parts, decreasing selectivity and increasing background noise (and radio-toxicity). Radionuclides with long half-lives may also cause biological damage. It is also important to limit the production of metabolites, especially those labeled with radionuclides. Metal radionuclides require knowledge of the coordination chemistry of their metal ions to optimize their choice of ligands from the perspective of their chemistry [17]. The chelator and linker are often combined into one molecule, known as a bifunctional chelator. The bifunctional chelator is attached to the vector by a linker or spacer, and is a molecule that contains the appropriate donor groups for the formation of a coordination compound with the radionuclide. Radiometals are the only ones that apply to this concept, and to ensure that no interference with the interaction with the target is caused by the coordination of the metal, the linker is positioned separately from the bioactive part of the molecule.

Innovative radionuclides and chelators are increasingly in demand in nuclear medicine. This is because the physical characteristics of radioisotopes and the biological characteristics of compounds are not always compatible with each other. A number of radionuclides, in fact, have a long half-life compared to targeting vectors (e.g., antibody fragments, small-molecule peptides that bind specific membrane receptors that are abundant on the surface of target tumor cells), which makes radioactive labeling with these radioisotopes incompatible [18]. Therefore, the goal is to find radioisotopes with a half-life compatible with the biomolecule carrier and optimize a chelator that is chemically compatible with the radionuclide to stabilize the radiopharmaceutical [19]. A major challenge in optimizing novel radiopharmaceuticals concerns the decay time and the limited use of some radioisotopes used in some assays [20]. This concerns the method of administration of these radionuclides for SPECT diagnostic use, such as, for example, ^67^Ga (half-life time 78 h), which is administered as Ga^3+^ citrate, which hydrolyzes slowly and is transported in the body by transferrin [21]. The long half-life of ^67^Ga allows its use even at very long distances from the place of production for the imaging of inflammatory processes and tumors. The use of the ^68^Ga isotope is sometimes preferred in the PET technique. This is because on the one hand, it uses the same 3+ ion family chemistry that they have as the only stable oxidation state Ga^3+^ and forms more stable structures with smaller macrocycles, type NOTA, than with DOTA. On the other hand, it also has the advantage of having a much shorter lifetime of about 68 min [22]. Thus, the goal is to develop radiopharmaceuticals that have radioisotope decay times comparable to biological half-lives [19].

Depending on other parameters, such as the depth of the target tissue and the resolution of the PET image, the energy of the particles emitted by each radionuclide can vary [23]. Optimal image quality depends on the energy of the particles emitted [24]. Table 1 shows the physical characteristics of all the radionuclides mentioned in this manuscript. Moreover, the goal of radionuclide production is to achieve maximum quality and purity of the radioisotope, which is directly connected with the safety profile and, therefore, image quality [25].

In radiotheranostics, the use of one radioisotope or a pair of radionuclides to mark the same targeting vector for a dual diagnostic and therapeutic purpose, is a hot research topic, aiming to optimize the use of a single drugs that perform two distinct functions, visualization and treatment through the exchange of specific radionuclides [26]. A theranostic pair may consist of two isotopes of the same element or two different elements with similar chemical characteristics. In the latter case, we report the use of ^68^Ga for PET or ^111^In for SPECT [27], followed by radionuclide therapy with lutetium-177 (^177^Lu) [28], yttrium-90 (^90^Y) [29] or actinium-225 (^225^Ac) [30]. Copper-64/copper-67 (^64^Cu/^67^Cu) [31] is an example of a radiotheranostic pair involving two isotopes of the same element, along with iodine-123-124/iodine-131 (^123/124^I^/131^I) [3], yttrium-86/yttrium-90 (^86^Y/^90^Y) [26], terbium isotopes [27], and scandium isotopes as well.

^64^Cu [17], ^67^Cu ^131^I, ^177^Lu, and ^47^Sc [3,17,31,32,33,34] are the most promising theranostic radionuclide examples. In order to improve the therapeutic efficacy of an ideal radionuclide for TRT, as well as consider the cross-fire effect and the complexity of DNA damage [35,36], which are partly correlated, it is necessary to study intercellular communication between healthy and unhealthy tissues, as well as the dosimetric aspect, through in vitro studies and dose-response curves.

In this regard, large tumors with heterogeneous vascularization and necrotic areas, should be considered, as they respond differently to radionuclides.

For radionuclide production sites, readings of [37,38] are recommended.

## 3. Radiopharmaceutical, Radiometric Counters, and Preclinical Imaging Techniques

**Figure 3:**
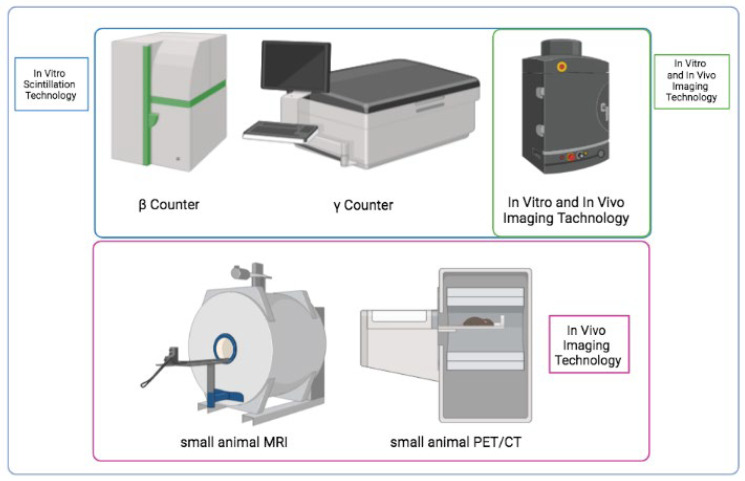
In vitro and in vivo systems for preclinical evaluation of radiopharmaceuticals.

Radiometric counters and imaging techniques (Figure 3) are commonly used for the uptake evaluation of radiopharmaceuticals administered in preclinical models (in vitro and in vivo, respectively).

A radiotracer can detect processes such as local blood flow, cellular metabolism, the expression of receptors in various cell types, neurotransmitter activity, trafficking and cellular homing, tissue invasion, and programmed cell death [39]. PET/CT and SPECT/CT devices in combination mode [40] allow pathological investigation of metabolism and morphology of organs, in addition to assessing treatment response [41]. The results of in vitro and in vivo preclinical studies are crucial to establish selectivity, specificity, and safety for each drug. These studies are also crucial for testing novel radiopharmaceuticals [42].

In preclinical research, new molecules are tested on mice, which are ideal for correlating results to human conditions [24]. However, it is necessary to conduct genotoxicity and cytotoxicity tests in vitro, before applying the drug to live animals. This is both to protect animal welfare and to identify the lowest concentration of the drug before applying it to in vivo models [25]. Using radioactive isotopes involves many risks for the operator, and radiation protection equipment and protocols are not available in all laboratories. As a result, many research groups use fluorescent compounds or other proxies instead of radionuclides for radiopharmaceutical in vitro testing [26]. However, even if this is useful in tracing the specificity and distribution of the drugs under examination, it does not provide any information on the biological activity of the radionuclides themselves. Consequently, several researchers have developed direct methods for detecting radiopharmaceuticals in vitro [43] by directly measuring the radiation (β, γ, or other) emitted by radionuclides through suitable radiometric counting devices. It has been shown that these methods have significant advantages, as they enable researchers to conduct direct counts of radioactive molecules. For radiopharmaceuticals to be properly translated, preclinical testing is required (both in vitro and in vivo), followed by clinical testing [44,45]. This work focuses on in vitro testing and excludes all other phases of radiopharmaceutical research. For detailed applications related to radionuclide imaging techniques and in vitro bioassays, please see [46]. For quantitative assessments of radiopharmaceutical biodistribution by in vivo assays, please see [47].

In vitro radioactivity experiments directly on cellular models using radiometric counters, represent the preliminary and preparatory steps for in vivo experiments [48]. In vitro studies with radiopharmaceuticals on cells can also be a useful method for scaling up more sophisticated counting methods, as PET scanners do not have the ability to correctly identify different coincidences, involving two 511 keV annihilation photons, and relative coincidences between an annihilation photon and a γ prompt, due to the fact that the energy of the γ prompt falls within the range of the scanner’s energy window.

Although the measurement is affected by tissue attenuation, some of these errors can be corrected to reduce their order of magnitude. This leads to a better resolution of the images. For this purpose, increasingly performing in vitro studies using radiometric counters could offer the opportunity to create new protocols to set and use a narrower energy window, capable of making it possible to improve the acquisition of both counts and images for isotopes that emit high-energy γ radiation in addition to positrons. Downstream, this option could reduce the background of images acquired with PET isotopes that emit high-energy γ radiation in addition to positrons [49]. Radiometric counters are radioactivity detectors. Two categories, namely β-counters and γ-counters, are commercially available. In this study, we will consider the β- and γ-counters, of which the designation refers to their ability to detect radionuclide emission, respectively, βand γ radiations. In addition to the detectors discussed in this review, there are several other types of detectors that can be used in other radiopharmaceutical and radiochemical applications [50]. 

The detection of alpha particles requires different considerations, because, considering that the range of action of alpha particles is very small, the detection and quantification of alpha particles in in vitro assays in radiobiology studies are generally performed through other types of analysis methods [51,52], which help avoid underestimation errors.

### 3.1. β-Counters Overview

**Figure 4:**
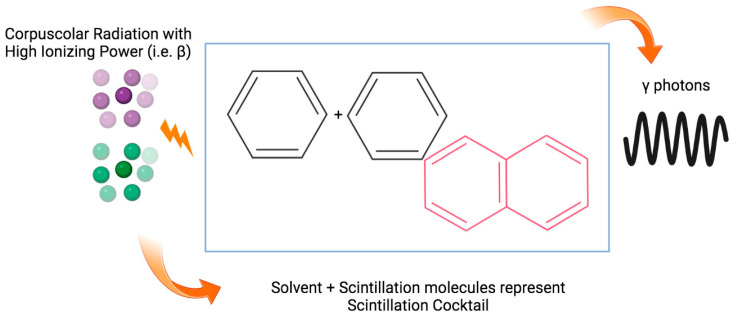
Mechanism of energy transfer in liquid scintillation counting.

β-counters are instruments used to calculate the radiation from β-emitters. An unstable nucleus emits fast electron beams when a neutron is converted into a proton by the emission of an electron with a negative charge (β−) or, conversely, when a proton is converted into a neutron by the emission of a positively charged positron (β+). These radiometric counters, also known as liquid-phase scintillators, are commonly used to determine the activity of β-emitters. The latter description is based on the fact that a cocktail of organic solvents and solutes is added to the sample, in order to detect radioactivity [53]. Solvents and solutes capture the energy of the β particles and convert it into a quantifiable parameter. The principle of this system is based on the idea that the β particles transfer some of their energy to the scintillation cocktail during their emission in the device [54,55]. In a few nanoseconds, the excited molecules that are part of the scintillator release this energy through light quanta [56]. In commercial scintillation cocktails, the energy is transferred from the primary scintillator to the secondary (wavelength-shifting) scintillator, releasing photons with wavelengths between 400 and 420 nanometers (see Figure 4). As a result of the sample being introduced into the liquid cocktail, the signal is quenched or reduced when part of the energy emitted by the radioisotope is not adequately captured by the photomultiplier tube of the counting instrument. To have accurate measurements, a correction must be applied to the counts due to the lower energy of the counts, which depends on the nature of the sample [57]. A second characteristic of the cocktail is that it contains solutes that absorb the energy of the photons produced by the solvent. These solutes then reemit them at a higher wavelength, which is captured by the photomultiplier more efficiently. By utilizing a dedicated system [58], the photomultiplier converts light quanta into electrical pulses. Using this strategy has the advantage of allowing for the use of radioisotopes with a different energy and a very long half-life for experiments directly on cells. There are several types of scintillators currently available in addition to liquid-phase scintillators, based on similar principles. As defined in [59], solid scintillators are classified by their different properties (such as the amount of energy converted into photons, response times, wavelength ranges emitted by photomultipliers and used in the product). In addition to this, to obtain an optimal counting statistic, it is important to consider a parameter, which, although not as dependent on the experimenter, is an important characteristic of the γ-counter instrument, namely the constant temperature of the photomultiplier. The photomultiplier can emit electrons even without a scintillator, due to temperature changes, which may result in an increase in background noise [60].

### 3.2. γ-Counters Overview

The γ-counter is designed to detect the activity of radiation from γ-emitters. The speed of γ rays is comparable to that of light, and they are considered electromagnetic radiation [61]. Specific counters based on inorganic substances, such as sodium iodide (NaI) crystals with traces of thallium, are commonly used to detect γ rays [62]. In response to an excitation with γ radiation, these crystals emit light with a wavelength of about 410 nm. In particular, γ rays are randomly generated by the γ-emitter radioisotope, which hits the thallium-activated sodium iodide crystal, and when this occurs, their energy is converted into light quanta and photons [59]. As a result, these photons are directed toward a quartz window connected to the photomultiplier, which emits an electrical impulse every time it is excited by a photon, with a frequency proportional to the radioisotope activity and an intensity proportional to the incoming photon energy [63,64]. Another technical feature of the crystals in terms of efficiency is the ratio between the actual activity of the radioactive source (expressed in disintegrations per minute—dpm) and the number of current pulses (expressed in counts per minute—cpm) as the output of the photomultiplier [56]. Efficiency values depend on the crystal size and other factors related to the radiation energy. Consequently, the size of the crystal acquires importance when it comes to signal acquisition, in order to achieve high efficiency. This is because photons generated by the decay of radioisotopes that emit radiation must transfer all of their energy to the crystal. Additionally, a constant voltage must be present in the photomultiplier, in order to accelerate electrons between two photodetectors [65]. As part of the counter, a signal analyzer is connected. This analyzer amplifies the current pulses, weighs the pulses coming from the high energy range, reduces background noise, and separates the different γ emissions [33].

## 4. Validation of Radiopharmaceuticals In Vitro by γ-Counter

### 4.1. Detection of ^64^Cu Compounds in Cells

^64^Cu is well studied both for diagnostic and therapeutic purposes, which makes it an excellent candidate for theragnostic purposes. The ^64^Cu has a half-life of 12.7 h and decays into ^64^Ni with positron emission (β+ = 17.9%, Emax = 660 keV, E average = 288 keV) or electron capture (EC = 43.1%, E = 1675 and 1346 keV), or decays into ^64^Zn, with the release of electrons (β^−^ = 39.0%, E = 190.2 keV) [34]. Copper exists in two oxidation states: Cu (I) and Cu (II). The first is less dominant than the second. Copper is generally more stable in vivo when conjugated with macrocyclic chelators [66]. Moreover, copper is usually bound to functional groups containing nitrogen, as it is found in compounds with coordination numbers ranging from four to six [67].

Hypoxia is a condition that occurs when a very low concentration of oxygen exists within the microenvironment of cells. Many forms of cancer, particularly the most aggressive types, are affected by this condition [68,69,70]. Since hypoxia in tumor tissue is crucial in predicting the type of response to oxygen-dependent therapies, and since Cu (II) undergoes a reduction to Cu (I) in hypoxic tissue and remains confined within, while this does not happen in normoxic tissue, this behavior allows imaging and diagnosis of the tissue where hypoxia is present [69], and can be exploited in imaging techniques.

As a result of poor perfusion in hypoxic tumors, conventional chemotherapy compounds are ineffective at delivering them to the tissue. Additionally, the lack of oxygen does not allow conventional radiotherapy to create free radicals that would cause the death of target cells in downstream steps. It is also known that cancer cells are more likely to undergo frequent genetic mutations under hypoxic conditions. This leads to more aggressive behavior and the ability to spread faster [71].

Human bodies contain an abundance of copper, which is essential for many biological functions. Copper levels usually range from 1.4 to 2.1 milligrams per kilogram [72]. It has been shown that copper levels are altered in a number of acquired and hereditary pathological contexts, such as cancer. There is an association between an increase in copper concentration in pathological conditions, and the progression of tumors and metastases. A number of cancerous tissues have shown overexpression of human copper transporter receptor 1 (hCTR1), for example, breast, lung, glioblastoma, and prostate cancer [73,74,75]. Copper is internalized within the cell through various enzymatic processes, such as CTR1, which allows the metal to penetrate the cell, and ATOX1, which allows the metal to enter the nucleus and is associated with cancer development [76]. Thus, this radionuclide has therognostic properties, which means that it does not cause toxicity to normal cells [77], but does cause cytotoxic and genotoxic effects on cancer cells.

In this study, Zheng Luo et al. [78] investigated two nitroimidazole derivatives, including [^64^Cu]-BMS2P2, that were able to target hypoxia in vitro, comparing their results to those of [^64^Cu]-BMS181321. [^64^Cu]-BMS2P2 uptake was evaluated by a γ-counter in epithelial tumorigenic cells under hypoxic and normoxic conditions, using [^64^Cu]-BMS181321 under hypoxic conditions. It was found that [^64^Cu]-BMS2P2 is a potential useful tracer of hypoxia for PET imaging. The highest level of cellular absorption of [^64^Cu]-BMS2P2 in a hypoxic environment was observed after one hour.

Among the reasons explaining why ^64^Cu is a desirable therapeutic candidate is its ability to emit radiotoxic Auger electrons, as well as particles. This is particularly evident in tissue areas with low oxygen levels. The study conducted by Weeks et al. [79] examined the amount of internalization of [^64^Cu]-diacetyl-bis (N(4)-methylthiosemicarbazone) ([^64^Cu]-ATSM) in breast cancer cells under hypoxic conditions, and the possible therapeutic effect of this compound. Various doses of [^64^Cu]-ATSM, up to a maximum of 10 MBq/mL, were administered to the MCF-7 cell line in these studies. The tests were conducted at increasing levels of hypoxia. To estimate the accumulation of ^64^Cu in cells, the multilabel γ-counter Triathler (Hidex, Turku, Finland) was used. In order to build new targeted therapeutics, in vitro radiobiology studies with cells grown under hypoxia are essential. In addition to clarifying the selectivity of a radiopharmaceutical compared to those already available for clinical use, these studies also illustrate its safety.

As atherosclerosis-induced inflammation plays an active role in heart and brain pathologies, as well as a range of chronic pathologies [80], studies on atherosclerosis in cardiology are becoming increasingly in-depth. PET/CT and PET/MRI [81] are both capable of detecting atherosclerotic plaque while it is growing, allowing for a correct diagnosis. It has been found that vascular endothelial cell adhesion protein VCAM-1 and plaque macrophages, the best known non-invasive markers of early atherosclerosis plaque [82,83], can be detected by accumulating [^18^F]FDG (Fluorodeoxyglucose) in macrophages, as described in [84]. The current research effort, however, aims to reduce background signals in order to improve plaque imaging signals [83]. A potential biomarker of atherosclerotic plaque is the cyclic LyP-1 peptide (CGNKRTRGC), due to its interaction with p32 overexpressed on macrophages of the plaque [85]. Seo and colleagues [85] synthesized dendrimers 6-BAT(6-[p-(bromoacetamido)benzyl]-1,4,8,11-tetraazacy-clotetradecane-N,N″,N‴,N′′′′-tetraacetic acid) and dentrimers-FAM (carboxyfluorescein or fluorescein amidite). An in vitro saturation-binding assay was performed using decreasing concentrations of [^64^Cu](LyP-1)4-dendrimer incubated with a fixed concentration of p32 protein. The radioactivity of the sample was measured by a γ-counter (PerkinElmer Life Sciences). It was discovered that with this in vitro study, the binding affinity of the target domains could be increased, and that (LyP-1)4-dendrimer could be exploited in future studies to improve nanoparticle therapies.

It was shown that the γ-counter can also be an efficient tool for measuring the immunoreactivity of immunoconjugates labeled with ^64^Cu. For example, Navarro et al. calculated the radioactivity of solutions containing antibodies labeled with ^64^Cu. This step was essential for the subsequent in vivo studies conducted on the syngeneic multiple myeloma model [35].

Currently, the PET imaging radiopharmaceuticals used in the clinical setting to detect amyloid β (Aβ) peptide aggregations in the brains of Alzheimer’s patients contain only ^18^F [18,36,37,38] and ^11^C, which, unfortunately, both have too-short half-lives. One of the fields of application of research on the optimization of radiopharmaceuticals labeled with ^64^Cu is to find bifunctional chelators for this radioisotope that are useful for detecting Aβ plaques. Nilantha, et al. [39] has studied these mechanics in vitro and in vivo using triazacyclonane and 2,11-diaza [3.3]-(2,6)pyridinophane(N4) macrocycles, as well as 2-phenyl-benzothiazole segments, to create chelated bifunctional structures. The in vitro binding study with ^64^Cu-radiolabeled complexes administered to Aβ40 fibrils in multiwall wells allowed for the calculation of the related radioactivity, through a Gamma 8000 counter containing a NaI crystal (Beckman Instruments, Inc., Irvine, CA, USA). This process was useful to fine-tune the subsequent steps in mouse models of Alzheimer’s.

Many tumors overexpress the transferrin receptor on the cell membrane, for example breast, colon, prostate, and cervical cancers [40,41]. The ligand of this receptor is transferrin, which is internalized in the cell through a well-defined process of endocytosis as soon as it comes into contact with the receptor. This ligand–receptor complex is stronger when transferrin is associated with iron (holo-transferrin). In view of this, the latter form may be a viable therapeutic target for the aforementioned tumors [42]. Kang et al. [86] proposed novel forms of chelators for ^64^Cu, based on NE3TA (7-[2-[carboxymethyl)amino]ethyl]-1,4,7-triazacyclononane-1,4-diacetic acid), named N-NE3TA and C-NE3TA. Thus, affinity and internalization studies of the radio complex consisting of NE3TA-transferrin and ^64^Cu (20 μCi/μL) were conducted on the PC3 human prostate cancer cell line, and the percentage of cellular uptake of the ^64^Cu-labeled transferrin conjugate was evaluated through Wallac Wizard 3″ γ-counter. Radiolabeled complex binding affinity is a prerequisite for biodistribution experiments on murine models in vivo. This study was critical to understanding which radiolabeled compound was absorbed most readily by a specific tumor cell line. As a result, in this case the radiometric analysis made the preclinical tests much more consistent from a translational research perspective. The study conducted by Alidori et al. [44] is very interesting. Here, four novel ^64^Cu-labeled complexes, based on 1,2,4-triazole, 3,5-dimethylpyrazole, 1,3,5-triaza-7-phosphaaamantane, and tris(hydroxy-methyl)phosphine, were tested in vitro after synthesis and characterization. The goal of this work was to verify if monophosphinic ligands labeled with ^64^Cu could be optimized for the construction of a radiopharmaceutical. Regarding the in vitro component, this aim has been partially achieved, using four [^64^Cu]-labeled compounds (5 μCi) on EMT-6 mouse mammary tumor cells, and, through a γ-counter, the amount of tracer associated with the cells was calculated. All four newly developed compounds showed a higher uptake level than the control.

In the oncology field, the behavior of epithelial integrin ανβ6, overexpressed on the cell membranes, is known to be a promoter of tumor invasion and progression [87,88], and this often indicates a poor prognosis for the survival of cancer patients (oral squamous cell, pancreatic, cervical, and non-small lung cancer cells) [89,90]. Therefore, it is considered a biomarker for aggressive tumors. Developing better chelators for ^64^Cu for inclusion in radiopharmaceutical formulations for antagonists to this biomarker, is an innovative idea. The authors, Huynh, et al. [91], studied the in vitro and in vivo properties of ^64^Cu-labeled complexes, composed of peptides (A20FMDV2) derived from foot-and-mouth disease viruses, which were bi-terminally PEGylated and conjugated to DOTA (S-2-(4-isothiocyanatobenzyl)-1,4,7,10-tetraazacyclododecane-tetraacetic acid) and PCTA (3,6,9,15-tetraazabicyclo[9.3.1] pentadeca-1(15),11,13-triene-4-S-(4-isothiocyanatobenzyl)-3,6,9-tetraazacyclododecane-tetraazacyclododecane-tetraazabicyl)pentadeca-1),11,13-triene-4-s)-triacetic acid). In this study, internalization and saturation-binding assays were performed on cell lines overexpressing the aforementioned integrins, such as CaSki epidermoid cervical cancer cell line, and on human pancreatic cancer BxPC3 cell, using 0.1 μCi of ^64^Cu-labeled compounds. In order to determine the radiometric activity, Beckman Gamma 8000 counters, containing NaI, crystals were used (Beckman Instruments, Inc., Irvine, CA, USA). It was possible to extrapolate quantitative data relevant to pharmacokinetics from in vitro assays, which were then utilized in preclinical testing.

Several peptidomimetics have been identified as being able to bind to transmembrane proteins in some types of melanomas, such as [^64^Cu]-LLP2A for the optimization of an effective radiotracer for PET imaging [92]. It is well known that VLA-4, a cell surface integrin that induces angiogenesis and the development of primary metastases in melanomas, can be considered a target for imaging and therapy diagnosis in the case of melanomas that develop primary metastatic niches [19]. A study conducted by Bellavia et al. [92] used increasing concentrations of [^64^Cu]-LLP2A in their experiments. Assays for measuring cell-bound radioactivity were conducted via γ-counting (PerkinElmer 2470 WIZARD2, Waltham, MA, USA), to evaluate cell binding and internalization of [^64^Cu]-LLP2A (12 μCi per well of a 12-well multiwell) on BPR cells overexpressing VLA-4. Radiopharmaceuticals were tested on these cells, since they are more similar to human cells. Based on these in vitro tests, the selective nature of [^64^Cu]-LLP2A was established against therapy-resistant tumors, thus making it a viable candidate for use as a theranostic agent.

One of the major challenges in radiochemistry is the development of bifunctional chelators for ^64^Cu, that prevent Cu(II) from dissociating from the ligand, making it suitable for in vivo imaging and therapeutic applications. Brett A. et al. [93] assessed in vitro and in vivo the stability of azamacrocycle-based chelators for ^64^Cu, added to dipeptides when used to target prostate-specific membrane antigen (PSMA) in comparison to picolinate chelate complexes. This study was conducted on BM-Sc3 PSMA-expressing cancer cells that were incubated with a maximum dose of 36.0 μCi of radiolabeled complexes, which were then analyzed. In the following step, cell-binding assays were conducted using the γ-counter. Additionally, the most effective chelators for ^64^Cu were tested in vivo, allowing for the selection of azamacrocycle-based chelators as the most stable. It has also been reported that studies on PET radiotracers, based on high-affinity antibodies or peptidomimetic compounds (named affibody), such as anti-HER2 affibody, are becoming increasingly promising. According to a study published by Qi et al. [94], an anti-HER2 affibody can be used to detect HER2-positive tumors. As part of this study, the ZHER2:342 antibody was radiolabeled with ^64^Cu and tested on the SKOV3 ovarian adenocarcinoma cell line. By using 74 kBq of [^64^Cu]DOTA-Cys-ZHER2:342 as the probe, this cellular line underwent absorption and saturation tests through a PerkinElmer 1470 automatic γ-counter (Waltham), and a high degree of selectivity and specificity of the radiotracer was observed.

### 4.2. Detection of ^68^Ga Compounds in Cells

A total of 40 gallium radioisotopes exists, three of which are commonly used in nuclear medicine (^66^Ga, ^67^Ga, and ^68^Ga). Of these, ^68^Ga is particularly suitable for PET imaging [94] of a wide range of tumor types, particularly neuroendocrine tumors [95]. There have been a variety of preclinical studies on the development of ^68^Ga tracers that have been applied to the detection of malignant tumors, functional tissue performance, neurological conditions, cardiac imaging, and rheumatoid arthritis, among others. In contrast to other cyclotron-produced radionuclides that have short half-lives, ^68^Ga is an easy-to-use, safe, and inexpensive source of PET radiotracers, that can be conveniently generated with a radionuclide generator that has a shelf-life of nearly one year. ^68^Ga is produced by radioactive decay, through electronic capture (EC) of ^68^Ge (half-life of 270.95 days) [96]. In turn, ^68^Ga decays have a half-life of 67.71 min, mainly generating ground state ^68^Zn.

^68^Ga(III) has unique characteristics that make it an excellent radiotracer for labeling biomolecules with a low molecular weight [97], such as antibody fragments, oligonucleotides, and peptides. As a result, this radioisotope can be used for in vivo imaging of biological processes, providing a level of radioactivity that permits the acquisition of high-quality images, short-term scanning, and radiation dose reduction at a minimum. In addition to its interesting physical properties and the availability of its own generator [98], ^68^Ga provides an excellent opportunity for the development of novel PET tracers. As a result of the availability and commercialization of ^68^Ge/^68^Ga generators [99], it is also possible to design a variety of ^68^Ga radiopharmaceuticals. This system allows direct access to a short-lived PET radionuclide within the nuclear medicine department for up to one year, in areas where cyclotrons are unavailable. Additionally, ^68^Ga coordination chemistry makes it an attractive candidate for PET. Since Ga (III) is a bioisoester of the iron cation, it is associated strongly with the biological transporter of iron [100,101]. Additionally, gallium-chelating complexes are sufficiently inert to transchelation by this biomolecule for proper efficacy in in vivo applications [102], eliminating the risk of exchanging a metal with an organic residue as a result of the process. By altering a molecule’s structure, the desired functions can be altered. For chemical stability, the development of compounds labeled with ^68^Ga requires bifunctional ligands, including macrocyclic compounds with nitrogen heteroatoms, such as chelates like DOTA and NOTA. As a theranostic radionuclide, ^68^Ga also has the potential to be applied in conjunction with other diagnostic radionuclides, such as ^90^Y and ^177^Lu [101]. 

Several studies have examined the potential role of [^68^Ga]citrate in the diagnosis of bone infections [103]. The studies examined infected animals and pathways associated with radiopharmaceuticals [104]. Some groups have demonstrated the accuracy of the tracer in cases of inflammation, for example [105]. However, they were not detected in experiments evaluating inflammation in animal models. In parallel, some works have focused on the large-scale production of this tracer [106].

Clinical imaging of bone metastases using [^68^Ga]-EDTMP [107] is a hot topic in the medical field. Compared to the ^18^F-fluoride, the advantage of using the ^68^Ga-labeled compound, particularly [^68^Ga]-EDTMP, was reduced, since it was not highly effective for the diagnosis of bone cancer. The result has led researchers to investigate more promising imaging probes for this cancer target. A novel class of gallium-labeled bone-imaging agents based on oligo-aspartic fractions has been developed, due to their high affinity for hydroxyapatite [108].

There have been many developments in recent years regarding DOTA-conjugated somatostatin derivatives (SSTs) [109], such as TOC, NOC, and TATE that are labeled with ^68^Ga for the diagnosis, planning, and monitoring of neuroendocrine tumors (NET). ^177^Lu and ^90^Y are therapeutic radionuclides that form stable complexes with the DOTA chelate and can be used as both therapeutic and diagnostic agents for diagnosis–therapy follow-up. [^68^Ga]-DOTATOC [110] was the first tracer used to detect malignant tumors, which was highly specific for SSR2 and SSR3, but less specific for SSR5. This method is extremely accurate at detecting NET, meningioma, malignant thyroid tumors, prostate cancer, and many other cancers [111]. However, there have been some problems with DOTATOC imaging in terms of showing false positive results for non-tumor tissues in the pancreas, pituitary gland, and chronic inflammatory conditions [112].

Another SST ligand developed for SST PET receptor imaging is [^68^Ga]-DOTATATE. [^68^Ga]-DOTATOC and [^68^Ga]-DOTATATE are equally suitable for staging and selecting patients for [^177^Lu]-DOTATATE peptide receptor radionuclide therapy. The advantages of [^68^Ga]-DOTATATE include its easy distribution and excretion by healthy organs [113], along with its ease of manufacture and quality control using automated and semi-automatic methods [114]. There are also many other studies on NET that have shown the high sensitivity and specificity of [^68^Ga]-DOTATATE [115]. The results of preliminary studies indicate that [^68^Ga]-DOTATATE absorbs lesions more efficiently, even in patients with well-differentiated thyroid cancer [116], compared with [^68^Ga]-DOTANOC. Radiotracer specific for SST 2, 3, 5 [^68^Ga]-DOTANOC identified a significantly higher number of lesions in patients with NET than radiotracer specific for SST2 ([^68^Ga]-DOTATATE) [117]. In their study, Jin et al. used human pancreatic NET cells, such as BON1, pancreatic islet tumor QGP1, broncho-pulmonary neuroendocrine NCI-H727, and midgut carcinoid GOT1. The study examined whether 5-fluorouracil alone, or in combination with chemotherapy decitabine or tacedinalin had therapeutic results [6,118]. In addition, the binding properties of [^68^Ga]DOTATOC radionuclides, after ionizing treatment of cell lines, were investigated in advanced neuroendocrine tumors. Based on the already validated peptide receptor radionuclide therapy [6,119,120], the uptake of [^68^Ga]DOTATOC-labeled peptides was assessed using 4 kBq of radioligand and measuring the radioactivity using a γ-counter (PerkinElmer). Thus, it has been possible to develop a more effective method of treating neuroendocrine tumors by using chemoradionuclides and making them radiosensitive. It is critical to note that this in vitro study was an extremely crucial first step in preclinical research. Since neuroendocrine tumors are prone to metastasizing, systemic treatment is necessary [121].

As the most common form of mesenchymal tumor affecting the gastrointestinal tract, gastrointestinal stromal tumor (GIST) often affects young patients and has a short life expectancy of about three years or less. This tumor can be treated by using tyrosine kinase inhibitor drugs (TKI), which are capable of selectively targeting the oncogenic TKI and platelet-derived growth factor receptors (PDGF-Ra), along with surgical removal [122]. GIST researchers are seeking to increase the knowledge and application of targeted radiopharmaceuticals for molecular imaging, radionuclide therapy, and non-invasive characterization [123]. GIST has been shown to overexpress a wide variety of receptors, such as an abundance of somatostatin 2A (SST2A) receptors in Cajal neuronal cells [124], as well as an upregulation of neurotensin 1 receptors, that are present regardless of the GIST degree of mutation [125]. As a result of the identification of these overexpressed receptors, nuclear medicine applications, such as PET and SPECT, as well as radionuclide therapy, are increasingly using peptides that are specifically radiolabeled with trivalent radiometals [126]. In a study conducted by Paulmichl et al., [^68^Ga]-labeled peptides targeting SST2A, VPAC2, NT1, GRP, and CCK2 receptors, were studied on three different GIST cell lines representing varying degrees of mutation [127]. The difference in sensitivity to TKI-based treatment was then examined. In the context of peptides, previous studies [128,129,130,131,132] were used to select DOTA-conjugated peptides (1,4,7,10-tetraazacyclododecane-N,N′,N″,N‴-tetraacetic acid). For the DOTA peptides, highly inconsistent results were observed regarding membrane binding and internalization. A 60 min timepoint was chosen as a reasonable compromise between high cell binding and internalization and ^68^Ga radionuclide decay after 68 min. During this study, it was found that, in relation to the gastrin-releasing peptide receptor (GRPR), there were high specific bindings found, both for an agonist (AMBA) with high internalization and for an antagonist (NeoBOMB1) with enhanced membrane-binding activity, across all GIST cell lines, regardless of the level of TKI resistance. This is supported by the excellent targeting levels of ^68^Ga-NeoBOMB1 found in both animal tumor models and in human prostate cancer [132]. Therefore, this radioligand might be a promising candidate for GIST targeting, for improved imaging and monitoring of tumors resistant to TKIs, as well as new radionuclide-based treatments.

Siderophores are small molecules that have a high affinity for iron and a high ability to chelate it. They are originally produced by bacteria, fungi, and a few plants. Their role is to transport iron across cell membranes, thereby making iron available to nourish all cells. With this mechanism, iron is also more available in contexts where microorganisms would not be able to easily collect iron. In the medical field, engineered siderophores are used in the administration of drugs for the treatment of diseases such as thalassemia and other forms of anemia. In addition, they are used for the treatment of some forms of cancer, and in the imaging of cancer and infections [43,45,46,48,133]. For imaging *Aspergillus* spp. infection, the use of ^68^Ga as a marker of siderophores has shown promise [47], since gallium (Ga^3+^; ionic radius = 62 pm) is similar to iron (Fe^3+^; ionic radius = 64 pm) with a similar structural and coordination geometry. Several siderophores can be labeled with ^68^Ga, demonstrating that [^68^Ga]triacetylfusarinin C (TAFC) and [^68^Ga]ferrioxamine E (FOXE) can detect *Aspergillus fumigatus* in the presence of an infection in rats by PET imaging [134,135]. Thus, [^68^Ga]TAFC has been shown to be highly specific for *A. fumigatus* in in vivo studies [134]. The study by Petrik et al. [134] demonstrated the solidity and flexibility of imaging siderophores radiolabeled with ^68^Ga and ^89^Zr for the detection of Aspergillus. For the in vitro characterization of radiolabeled siderophores, protein-binding studies were performed with [^68^Ga]siderophores and [^89^Zr]siderophores through incubations in fresh human serum and in PBS (as a control) at 37 °C, at different time points. The determination of radioactivity was measured through an automatic γ-counter suitable for WIZARD2 radiometric detection (PerkinElmer, Waltham, MA, USA). Stability studies of [^89^Zr]siderophores showed some differences compared to [^68^Ga]siderophores [54]. In particular, [^68^Ga] and [^89^Zr]triacetylfusarinin C (TAFC) were found to be the most stable and with a higher binding affinity. Even though ^68^Ga and ^89^Zr are different chemical elements, their labeling has led to interesting investigations in imaging infections. This can be attributed to the fact that both elements can be acquired at varying times, ranging from minutes to days, respectively. In this way, these two radionuclides were selected for their different half-life and their same radioactive decay through EC/β+.

Cancers of the digestive system, such as stomach and colon cancers, are among the most aggressive since they are generally detected very late in the disease process. Inflammatory cells, including macrophages, make up the tumor microenvironment and are often responsible for the resistance of tumors to therapies [55]. There is a difference between tumor-associated macrophages [136] and other types of macrophages associated with inflammation due to the fact that, in addition to having a peculiar phenotype [137], they exhibit massive expression of the triggering receptor expressed marker on myeloid cells 2 (TREM-2) [138,139]. Chen et al. [56] identified COG1410, apolipoprotein mimetic peptides, as a ligand for TREM-2. Shi, Dai, et al. [140] conducted an experiment using [^68^Ga]NOTA-COG1410 as a specific radioligand targeting TREM-2, to distinguish tumors from inflammation. To do this, a binding assay and competitive-binding assay of [^68^Ga]NOTA-COG1410 on tumor-associated macrophages (TAM cells) were conducted. In vitro radioactivity was detected to calculate Kd and Bmax coefficients [141], evaluation parameters of drug–receptor binding, showing reasonable efficacy of the compound in treating digestive tumors. Kumar et al. [59] developed an in vitro test using [^68^Ga]NODAGA to label a compound made by a combination of vasoactive intestinal peptide and pituitary adenylate-cyclase-activating peptide receptors (known as VPAC receptor-specific peptide) on human BT474 breast tumor cells. The evaluation of cell binding and specific binding of 0.74 MBq of radiolabeled compounds was carried out using a Wizard2 2480 γ-counter (Perkin Elmer). This study demonstrated how the radiolabeling procedure is reproducible and that the [^68^Ga]NODAGA peptide is more selective in PET-imaging studies than other ^68^Ga chelators. 

Siyuan, et al. [61] exploited knowledge of bombesin as a homolog of the gastrin-releasing peptide, which is capable of binding to the gastrin-releasing peptide receptor, GRPR [62,64], highly expressed in prostate cancer cells, and in other cancers [63]. Literature data show that bombesin antagonists are less capable of being internalized. However, on the other hand, they are more useful when radiolabeled than agonists for in vitro studies [142,143]. In this context, Siyuan, et al. [144] performed a γ-counter comparative study, measuring uptake, internalization, and cell efflux in PC-3 prostate cancer cells, using [^68^Ga]NOTA-Aca-BBN7-14 and [^68^Ga]NOTA-PEG3-RM26 in PC-3 cells, at different times and concentrations. The in vitro studies, supported by the preclinical trial, showed that [^68^Ga]NOTA-PEG3-RM26-targeted antagonist probe is a more effective radiotracer for visualizing prostate cancer than its agonist counterpart. Considering that there are many imaging issues related to this organ, this result can certainly be considered promising. Sung-Hyun et al. [65] exploited glu-urea-lys derivatives as prostate-specific membrane antigen agents [145], conjugating them through thiourea with [^68^Ga]-labeled NOTA or DOTA. The results of the in vitro cell-binding assay, using 22Rv1 PSMA-positive human prostate cancer cell line, were obtained using a γ scintillation counter (DREAM G-10, Shinjin Medics Inc., Shinjin, Korea), making the use of these compounds promising for prostate cancer imaging. Finally, [^68^Ga]NOTA-GUL could be a promising radiopharmaceutical specific to prostate-specific membrane antigen.

### 4.3. Detection of ^125^I Compounds in Cells

Iodine ^125^I is an interesting radioisotope for imaging, as it is essential for in vitro validation of corresponding isotopes suitable for incorporation into radiopharmaceutical structures, such as ^123^I or ^131^I. The latter two isotopes are often used in the labeling of drugs that target cancers such as prostate cancer, uveal melanomas, and brain tumors.

Its half-life is 59.49 days and it decays with electron capture to an excited state of tellurium-125. This state is not metastable, but rather immediately decays with γ decay, with a maximum energy of 35 keV. Some of the excess energies of the excited states could be converted internally into ejected electrons (even at 35 keV), or X-rays (from electron bremsstrahlung), and into Auger electrons, which are produced at low energies of 50 to 500 electron volts [146]. In medical applications, internal conversion and Auger electrons cause only minimal damage to the outside of the cell that contains the radioisotope. Due to its relatively long half-life and low-energy photon emission that can be detected by γ crystal detectors, ^125^I is a preferred radioisotope for antibody labeling in radioimmunoassay and other γ-counting procedures that involve proteins outside the body.

The CXCR4 receptor is a receptor for chemokines which, when hit by its ligands, can activate multiple pathways, such as cell migration, hematopoiesis, and others. CXCR4 has the peculiarity of acting directly on the proliferation of non-hematopoietic cells [66]. This receptor is involved in stem cell activation and tissue regeneration, as it is connected to tumor growth. In the study by Schottelius et al. [147], the efficiency of human CXCR4 receptor (hCXCR4) and murine CXCR4 ortholog (mCXCR4) was evaluated in target cells both in vitro and in vivo through cyclo(-D-Tyr1-D-[N-hexyl-6-guanidino]Ala2-Arg3-Nal4-Gly5) labeled with ^125^I ([^125^I]CPCR4.3), as CPCR4.3 showed high affinity for mCXCR4 [147]. In this study, cell lines that grew in suspension (Daudi human Burkitt lymphoma cells, Jurkat human T-lymphocyte cells) overexpressing endogenous hCXCR4 and adherent cell lines, such as Eμ-myc 1080 mouse B-cell lymphoma cells overexpressing mCXCR4, were used for comparative-binding studies. The cells were incubated with 0.1 nM [^125^I]CPCR4.3, and the activity of free and receptor-bound radiopharmaceutical was measured by γ-counter (WALLAC WIZARD 3 1480). This in vitro study was critical for quantifying competitive binding through the calculation of IC_50_, i.e., minimum inhibitory concentration to inhibit 50% of a target. This parameter is essential when a novel radiopharmaceutical is under development, and thus the use of the γ-counter for in vitro tests is preliminary to the subsequent preclinical tests.

One of the most commonly discussed biomarkers of tumors is cyclooxygenase-2 (COX-2). The imaging probes that are currently being used on COX-2-positive tumors do not allow us to distinguish the tumor effectively from the background. Therefore, numerous studies are focused on the research and development of new molecular imaging probes for in vitro and in vivo tests on COX-2-expressing carcinomas. Absorption studies of indomethacin (Ind) derivatives labeled with ^125^I on COX-2-expressing cells were conducted [148]. In particular, HEK cells with inducible COX-2 expression and COX-1-positive HUVEC cells were incubated with 0.2 MBq/well of [^125^I]Ind derivatives. The measurement of radiopharmaceutical uptake by the cells was performed through the γ-counter (Wizard2, PerkinElmer). As demonstrated in this study, in vitro experiments are almost mandatory before animal model biodistribution experiments can be conducted.

In the field of breast cancer, novel biomarkers or targeting agents are increasingly being studied and investigated. A potential tumor-targeting agent is crotoxin (Crtx) [149], a polypeptide macromolecule present in the venom of *Crotalus durissus terrificus*. The peculiarity of this venom-isolated peptide is that it binds to the epidermal growth factor receptor (EGFR) [68], which characterizes breast cancer in more than half of patients with this disease. Since the aim of the research is to increase the development of increasingly performing radiopharmaceuticals for the diagnosis and treatment of specific cancers, Silveira et al. [69] analyzed the characteristics of Crtx labeled with ^125^I on Ehrlich ascites tumor cells (EAT cells), which, like breast cancer cells, express EGFR. In vitro experiments were conducted through receptor binding of [^125^I]Crtx and competition assays on EAT cells incubated with 1 × 10^−10^ M of [^125^I]Crtx. An automatic 1480 Wizard 3″- Wallac γ-counter was used to measure the bound radioactivity. After that, a range of various concentrations, from 3 × 10^−12^ to 2 × 10^−8^ M, of [^125^I]Crtx was administered to the cell line to perform the saturation-binding assay. By conducting these in vitro experiments, it was possible to analyze the pharmacokinetic profile of this radiotracer, including the concentration of ligand that inhibits 50% of the maximum specific binding (IC50) and the radioligand equilibrium dissociation constant (Kd).

One study [150] used an electrophilic substitution procedure to label pirarubicin with [^125^I]. The radiochemical yield of the labeled molecule was near 98%, and it consistently stayed over 90% for more than 20 h at room temperature, and when serum was present at 37 °C. It was determined in silico whether [^125^I] pirarubicin would bind to the DNA human topoisomerase II complex, which is its target. With a nuclei-to-cells ratio of 40 ± 1.2%, the in vitro tracer uptake by cancer cells was calculated by a γ-counter, and it reached saturation in about 3 h. The growth inhibition test demonstrated that the labeled compound’s antiproliferative activity was significantly stronger than that of unlabeled pirarubicin. Radiotoxicity increases the cytotoxicity of cancer drugs. The [^125^I] pirarubicin seems to preferentially accumulate in urinary bladder malignant tissues, according to in vivo results.

There is a branch of research that examines how to develop imaging probes that can maintain a high affinity for the receptor to be targeted. This is because imaging probes cannot activate all the tumorigenic mechanisms of downstream processes. This is the case with radioactive probes for diagnostic/therapeutic imaging for the secretin receptor highly expressed on gastric and hepatic tumor cells [41,151,152]. Anja et al. [153] performed in vitro experiments through radioligand-binding assay, using a scintillation counter (PerkinElmer, Rodgau, Germany) and a MicroBetaII plate-based scintillation counter (PerkinElmer, Rodgau, Germany) to evaluate the amount of bound radioligand on U2OS human osteosarcoma cell line. This work was especially relevant for potentiating secretin analogs for their use as radiotracers, for imaging and in vivo theranostics. Chao et al. [154] measured Ki, a parameter used in in vitro competition assays, in stably transfected HEK293 cells expressing human angiotensin Ι receptor-related protein J receptor, treated with [^125^I]apelin-13 together with different concentrations of Elabela fragments. Elabela fragments are endogenous ligands of human angiotensin Ι receptor-associated protein J receptor. These measurements were performed through PerkinElmer 2470 Automatic γ-counter WIZARD (Norwalk, CT, USA), allowing for finding analogues of Elabela fragments that had the highest affinity for angiotensin Ι receptor-associated protein J receptor. These results have been useful in designing both in vitro and in vivo effective strategies to treat cardiovascular pathologies. This is because these peptides are regulators of various processes in human physiology [155,156,157,158,159,160]. Potalitsyn et al. [161] performed saturation-binding experiments using different increasing concentrations of [^125^I]monoiodotyrosyl-Tyr2-IGF2 capable of binding IGF2 receptor, measuring radioactivity through Wizard 2470 γ-counter (Perkin Elmer) for the characterization of novel forms of insulin-like growth factors 2, particularly involved in memory and cognitive functions of the nervous system [162,163,164,165].

### 4.4. Detection of ^99m^Tc Compounds on Cells

Technetium-99m (^99m^Tc) is a metastable nuclear isomer of technetium-99, employed in biomedical imaging technologies, in particular in SPECT imaging [166] as a radioactive tracer. The uptake of radioactivity emitted by radiopharmaceuticals can be detected both with equipment suitable for cell plates and with equipment suitable for patients called γ-counters or γ-cameras, respectively [167,168]. The advantage of using this compound in clinical practice is that its half-life for γ emission is 6.0058 h (this means that 93.7% of it decays to ^99^Tc in 24 h). Because this isotope has a biological half-life of about a day, it can be used for quick scans, thereby limiting the patient’s exposure to radiation. Therefore, this isotope is purely diagnostic and is not suitable for therapeutic treatments [168]. In the literature, 99mTc is present in many works, both in vitro and in vivo.

Ideally, a drug delivery system should be safe, should not trigger immune responses in the body [169], should transport the compound of interest without leaking or exchanging homologues present in biofluids, and should deliver it to the cells, tissues, or organs of interest. Liposomes are widely used as vehicles for the administration of peptides, proteins, and other active substances for pharmaceutical and biochemical purposes [170]. In fact, liposomes are used to transport antibacterial and antiviral substances, as well as hormones, enzymes, nucleic acids, and antibodies [171]. However, they have the disadvantage of activating immune responses triggered by the complement system. An in vitro study performed in Turkey in 2020 [172] demonstrated that the [^99m^Tc]-labeled folate-targeted nanosized liposomes, as radiopharmaceuticals, produced an effective radiopharmaceutical on Lewis lung carcinoma (LLC1) cells for diagnosing a very common form of non-small cell lung cancer (NSCLC). The uptake rate of the radiopharmaceutical was assessed by measuring the radioactivity of the cells through a γ-counter (GE Healthcare, USA), using multi-well culture plates, and a comparative evaluation of cellular uptake of the fluorescence probe and [^99m^Tc]liposomal formulations was performed. The radiolabeling efficiency of liposomes was high and, in this context, the use of the γ-counter was essential, since the toxic side effects of chemotherapy drugs on healthy cells expressing fewer folate receptors than cancer cells had a decrease.

The condition of multi-drug resistance (MDR) occurs when a tumor becomes resistant to multiple therapies at the same time [173]. There are many causes; however, the overexpression of a gene known as MDR1 gives the cell a particular resistance [70]. Therapies capable of interfering with the harmful action of the MDR1 gene, which produces a protein capable of blocking the entry of the chemotherapy drug into the cell (thus protecting it from its action) are being studied. Other genes overexpressed in MDR tumors are those that code for ATP-binding cassette (ABC) transporters, such as P-glycoprotein (Pgp), multiple-resistance-associated protein 1 (MRP1) and others, in the plasma membrane [174,175]. The overexpression of these ABC transporters causes the energy-dependent escape of chemotherapeutic agents from the intracellular volume to the extracellular environment. MDR tumor cells show poor outcomes for patients as a result of their resistance to chemotherapy [176,177]. In nuclear medicine, [^99m^Tc]hexakis-2-methoxyisobutylisonitrile (MIBI) is an agent used in imaging of the parathyroid gland and the heart. MIBI, as a substrate for Pgp and MRP1, exhibits similar pharmacokinetics to chemotherapeutic agents such as doxorubin [178,179,180]. Several clinical studies have shown that MIBI is a valid predictor of chemotherapy response in non-small cell lung cancer [181,182,183,184]. Yumiko et al. [175] used [^99m^Tc]MIBI in vitro and doxorubicin in vivo to investigate how to optimally combine chemotherapy and radiotherapy to achieve the maximum synergistic effect in chemoradiotherapy, using non-small tumor cells and irradiation. To evaluate MDR suppression after γ-ray irradiation, [^99m^Tc]MIBI accumulation was performed in an in vitro study in human NSCLC cells. The dose rate of **γ**-ray irradiation was 0.84 Gy/min, and a radiometric counter (WIZARD “3” 1480, Perkin Elmer, Life Science) was used to monitor cellular accumulation of MIBI several days after irradiation. An increase in intracellular MIBI retention was observed 4 to 14 days after irradiation, highlighting the suppression of MDR in H1299/wtp53 due to irradiation. In addition, MDR suppression was found to be γ-irradiation dose dependent in terms of amount and duration, suggesting its key role in clinical chemoradiotherapy.

The concept of multimodal imaging defines a biomedical imaging system that uses optical, radioactive, and magnetic properties simultaneously. This imaging technique consists of PET, optical fluorescence, MR spectroscopy, bioluminescence, and SPECT. Using different imaging methods, it is possible to see different aspects of the body, providing a more comprehensive view of a specific tissue in the human body. Through the multimodal image, almost anything present in a specific tissue can be visualized. This includes its exact position, size, and metabolic activity, in both the district of interest and surrounding tissues. This is in order to assess possible alterations or dysfunction of these tissues caused by pathological conditions such as a tumor or any other medical complication. In cancer surgery, a dual nuclear and fluorescent imaging agent, targeting cancerous tissue, could be useful in determining the progress of the cancer. This could be useful in determining the size and location of the tumor in the body.

In addition to anatomical distortion, optical imaging can provide real-time distinction between healthy and diseased tissues during surgery [185]. It is essential to not only remove the tumor completely, but also to monitor how surgery progresses [186].

Kim et al. [71] developed [^99m^Tc]folate-Gly-His-Glu-Gly-Glu-Cys-Gly-Lys(-5-carboxy-X-rhodamine)-NH2 (folate-ECG-ROX) as a dual-modality imaging, to target tumor cells expressing the folate receptor. Simultaneous labeling with ^99m^Tc and ROX could provide nuclear and fluorescent images. The diagnostic performance and surgical use of [^99m^Tc]folate-ECG-ROX as a dual-modality tumor imaging agent were also evaluated in a mouse model. A substantial and specific affinity of [^99m^Tc]folate-ECG-ROX for the folate receptor emerged from in vivo and in vitro studies. After the synthesis and characterization of folate-ECG-ROX, radiolabeling of folate-ECG-ROX with ^99m^Tc was performed. Studies on its binding affinity and cellular uptake in vitro were conducted on KB human papilloma and HT-1080 fibrosarcoma cell line human epithelial cells. The binding affinities of [^99m^Tc]folate-ECG-ROX for KB and HT-1080 cells were determined by saturation-binding experiments, by determining the total binding and non-specific binding at different concentrations of the radiolabeled ligand. In particular, KB and HT-1080 cells, respectively, expressing and non-expressing folate receptors, were seeded in multi-well plates with a concentration gradient of as much as 500 nM of [^99m^Tc]folate-ECG-ROX added to the wells for 1 h. For measurement analysis, the cells of each well were collected and a 1480 Wizard 3 γ-counter (PerkinElmer Life and Analytical Sciences, Wallingford, CT, USA) was used. The results of this work showed that the Kd of [^99m^Tc]folate-ECG-ROX for HT-1080 cells was estimated to be significantly higher than that for KB cells, demonstrating that [^99m^Tc]folate-ECG-ROX shows a high affinity for the specific uptake of the folate receptor, validated through confocal microscopy analysis. In the in vivo component, [^99m^Tc]folate-ECG-ROX was identified as a preoperative imaging agent, which improved diagnostic accuracy and as an intraoperative imaging agent for tumors expressing the folate receptor during surgery.

Concerning imaging techniques for prostate cancer, an evolution in the development of radiotracers has occurred. This evolution is aimed at making an early diagnosis of the disease, staging of the cancer, and evaluation of patients’ response to treatment [187,188,189]. The diagnosis of prostate cancer has a strong impact on therapeutic decisions and targeted treatments. For the development of a SPECT-based PSMA radioligand, various agents labeled with ^99m^Tc are present [72]. The developed PSMA-targeted ligands generally consist of small molecules based on the glu-urea-lys pharmacophore, which binds to the active site of the PSMA enzyme, inhibiting it [73]. Kusum, et al. [73] explored the possibility of ^99m^Tc-labeling of a targeted HBED–CC conjugated PSMA ligand, PSMA-11, by carrying out experiments to optimize radiolabeling parameters and quality control procedures. Stability studies of [^99m^Tc]PSMA-11, measurements of cellular uptake in vitro and the determination of the pharmacokinetic patterns in vivo were performed and finally, preliminary clinical studies using SPECT were accomplished. In vitro cell absorption was performed with prostate cancer cells, LNCaP (PSMA-positive) and PC3 (PSMA-negative), cultured in multi-well tissue culture plates and incubated in 8 pmol and 37 kBq of [^99m^Tc]PSMA-11 per well for 1 h. The radioactivity was measured using a NaI (Tl) γ-counter, followed by inhibition studies. The results of this study showed a high uptake of the radiotracer [^99m^Tc]PSMA-11 in PSMA-positive LNCaP prostate cancer cells, as compared with PSMA-negative PC3 cells. All this points to the receptor specificity of [^99m^Tc]PSMA-11, as the uptake of the radiopharmaceutical is significantly high in PSMA-positive LNCaP cells and significantly reduced during blocking studies. [^99m^Tc]PSMA-11, thus, represents a possible SPECT-imaging agent for prostate cancer, both for primary prostate cancer and for associated metastatic site detection and therapeutic monitoring.

Clinical imaging uses radiopeptides able to target many tumors, including cancers of the breast, intestine, brain, and pancreas [74,75]. Bombesin (BN), a neuropeptide isolated from frog, is very similar to human gastrin-releasing peptide (GRP), of which the receptors are overexpressed on the surface of cells in the early stages of carcinogenesis [76]. The similarity between these two peptides with less than 30 amino acids is based on their biological function and the fact that they differ only by an amino acid at the carboxy-terminal end [190]. Radionuclide labeling of bombesin exploits the highly specific bond between BN and GRP-R [77]. The development of peptides that can penetrate cells has become an increasingly hot topic. This is because the increase in efficiency of radiopharmaceutical transport within the cell has been a topic of intense research for years. One of the peptides that has been successful for the transport of nanoparticles, nucleic acids or proteins is the HIV-TAT peptide [191]. Santos-Cuevas et al. [192] evaluated the in vitro absorption kinetics of a new hybrid radiopharmaceutical, [^99m^Tc]N2S2-Tat(49–57)-Lys3-bombesin ([^99m^Tc]Tat-BN), on GRP receptor overexpressing tumor cells, comparing its cellular internalization with that of [^99m^Tc]BN. PC-3 cells from prostate cancer and MDA-MB231 and MCF7 cells from breast cancer were incubated five times (up to 24 h) with 200.000 cpm of [^99m^Tc]BN or [^99m^Tc]Tat-BN. Radioactivity was measured by an unspecified crystal scintillation detector through internalization and specific-binding assays. This study demonstrated how in vitro kinetic studies using a γ-counter are mandatory together with in vivo experiments, before moving onto clinical trials. Here, radiolabeled compounds, capable of crossing a lipophilic cell membrane, have been studied directly on the cells, in order to determine their level of internalization, and thus their specificity. A hybrid compound, such as the one used in this study, could potentially be used in in vivo experiments and for patients’ imaging for the treatment of prostate and breast cancer.

In the aforementioned articles, various types of γ-counter have been used to study in in vitro cellular uptake and drug–receptor interaction through the use of radiolabeled compounds with ^99m^Tc. Furthermore, these studies show the importance of these technologies and the preliminary nature that such studies possess in the preclinical setting. Clinical application to patients will follow this step. Currently, many molecules of innovative ligands and specific chelating agents are being studied. This is done in such a way as to make sure that the detection methods of ^99m^Tc-based radiopharmaceuticals, and radiopharmaceuticals in general, are more and more efficient.

## 5. Conclusions

In recent decades, advances in technology applied to cell culture models for in vitro studies have contributed to a better understanding of internalization and externalization mechanisms of radioactive compounds designed for personalized therapy through PET and SPECT imaging, and radiomics analysis. In nuclear medicine and radiobiology, instruments such as radiometric counters have made it possible to obtain more sophisticated and precise experimental evidence. As a result, these experiments can be translated into preclinical approaches, making these types of biological targeting studies much more technically accurate. Although all the works presented in this review aim to guide future researchers enrolled to work from a biological point of view on in vitro assays of radiopharmaceuticals conducted through radiometric counters, the limit of counting efficiency must always be considered due to the absorption of radiation by the sample that emits the radiation, for example. In fact, great care must be paid when using drugs labeled with radionuclides that have a low-emission energy, such as ^125^I [193,194].

Research in this area of imaging is becoming increasingly relevant to improve studies on the dosimetric quantification of PET tracers, to obtain more specific radiotracers for diagnostic use. In this way, it is possible to carry out a global characterization of a compound from a radiogenomics approach. This includes both in vitro and preclinical analyses. Finally, these radiometric counters will be increasingly indispensable in the future because, in addition to providing better performing results, they allow for a more prudent and responsible use of radionuclides applied to in vitro cellular assays for the development of therapeutic radionuclides.

## Figures and Tables

**Table 1:** Physical properties of commonly used imaging (PET/SPECT) radionuclides discussed in this review.

Radionuclide	T_1/2_	Mode of Decay *	Decay Product	Energy (keV)
^64^Cu	12.7 h	EC β^+^ (61.5%) β^−^ (38.5%)	^64^Ni ^64^Zn	278.01 190.74
^68^Ga	68.1 min	EC β^+^ (100%)	^68^Zn	836
^125^I	59.4 day	EC (100%)	^125^Te	35.5
^99m^Tc	6.06 h	IT(89%)	^99^Tc	140.511
^177^Lu	6.64 d	β^−^ (100%)	^177^Hf	148.8
^90^Y	64.05 h	β^−^ (100%)	^90^Zr	932.4
^225^Ac	9.92 d	α (100%)	^221^Fr	5830
^67^Cu	3 d	β^−^ (100%) γ (52%)	^67^Zn	185
^123^I	13.2 h	EC(100%)	^123^Te	159
^124^I	1003.2 h	EC β^+^ (100%)	^124^Te	366.8
^131^I	8.02 d	β^−^ (100%)	^131^Xe	191.6
^86^Y	14.74 h	EC β^+^ (100%)	^86^Sr	589

* Abundance in %; EC, electron capture; IT, international transition; TNB, thermal neutron bombardment.

## Data Availability

Not applicable.

## Abbreviations

SPECT	Single-photon emission computed tomography
TRT	Targeted radionuclide therapy
LET	Linear energy transfer
DNA	Deoxyribonucleic acid
BFC	Bifunctional chelator
PET	Positron emission tomography
CT	Computed tomography
hCTR1	Human copper transporter receptor 1
ATOX1	Antioxidant 1 copper chaperone
BMS2P2	2-nitroimidazole derivative
DOTA	S-2-(4-isothiocyanatobenzyl)-1,4,7,10-tetraazacyclododecane-tetraacetic acid
PCTA	3,6,9,15-tetraazabicyclo [9.3.1] pentadeca-1(15),11,13-triene-4-S-(4 isothiocyanatobenzyl)-3,6,9-triacetic acid
PSMA	Prostate-specific membrane antigen
NOTA	1,4,7-triazacyclononane-N,N′,N″-triacetic acid
NET	Neuroendocrine tumors
EDTMP	Ethylenediamine tetra(methylene phosphonic acid)
GIST	Gastrointestinal stromal tumor
TKI	Tyrosine kinase inhibitor drugs
PDGFR	Platelet-derived growth factor receptor
NT1	Neurotensin 1
SST2A	Somatostatin receptor
VPAC2	Vasoactive intestinal peptide receptor type 2
GRP	Gastrin-releasing peptide receptor
CCK2	Cholecystokinin receptors
AMBA	4-(Aminomethyl)benzoic acid
TREM-2	Marker triggering receptor expressed on myeloid cells 2
COG1410	Apolipoprotein E (apoE) mimetic small peptide
TAM	Tumor-associated macrophages
BON-1	Pancreatic neuroendocrine tumor cells
QGP1	Human pancreatic endocrine cell line
NCI-H727	Bronchial carcinoid cell
GOT1	Human small intestine NET cell line
GUL	Glu-urea-lys
CXCR4	C-X-C chemokine receptor type 4
IGF2R	Insulin-like growth factor 2 receptor
NSCLC	Non-small cell lung cancer
MIBI	Hexakis-2-methoxyisobutylisonitrile
[^99m^Tc]Tat-BN	[99mTc]N2S2-Tat(49–57)-Lys3-bombesin
